# Surveillance for *Borrelia burgdorferi* in *Ixodes* Ticks and Small Rodents in British Columbia^*^

**DOI:** 10.1089/vbz.2015.1854

**Published:** 2015-11-01

**Authors:** Muhammad G. Morshed, Min-Kuang Lee, Stephanie Man, Keerthi Fernando, Quantine Wong, Andrias Hojgaard, Patrick Tang, Sunny Mak, Bonnie Henry, David M. Patrick

**Affiliations:** ^1^ British Columbia Public Health Microbiology and Reference Laboratory, Vancouver, British Columbia, Canada.; ^2^ University of British Columbia, Vancouver, British Columbia, Canada.; ^3^ Centers for Disease Control and Prevention, Fort Collins, Colorado.; ^4^ British Columbia Centre for Disease Control, Vancouver, British Columbia, Canada.; ^5^ British Columbia Ministry of Health, Victoria, British Columbia, Canada.

**Keywords:** Lyme disease, Surveillance, Ticks, Mice, *Borrelia burgdorferi*, PCR

## Abstract

To determine the prevalence of *Borrelia burgdorferi* in British Columbian ticks, fieldwork was conducted over a 2-year period. In all, 893 ticks (*Ixodes pacificus*, *I. angustus*, *I. soricis*, *Ixodes* spp., and *Dermacentor andersoni*) of different life stages were retrieved from 483 small rodents (*Peromyscus maniculatus*, *Perognathus parvus*, and *Reithrodontomys megalotis*). *B. burgdorferi* DNA was detected in 5 out of 359 tick pools, and 41 out of 483 mice were serologically confirmed to have antibodies against *B. burgdorferi*. These results were consistent with previous studies, data from passive surveillance in British Columbia, and data from neighboring states in the Pacific Northwest, suggesting a continually low prevalence of *B. burgdorferi* in British Columbia ticks.

## Introduction

**FIG. 1.**
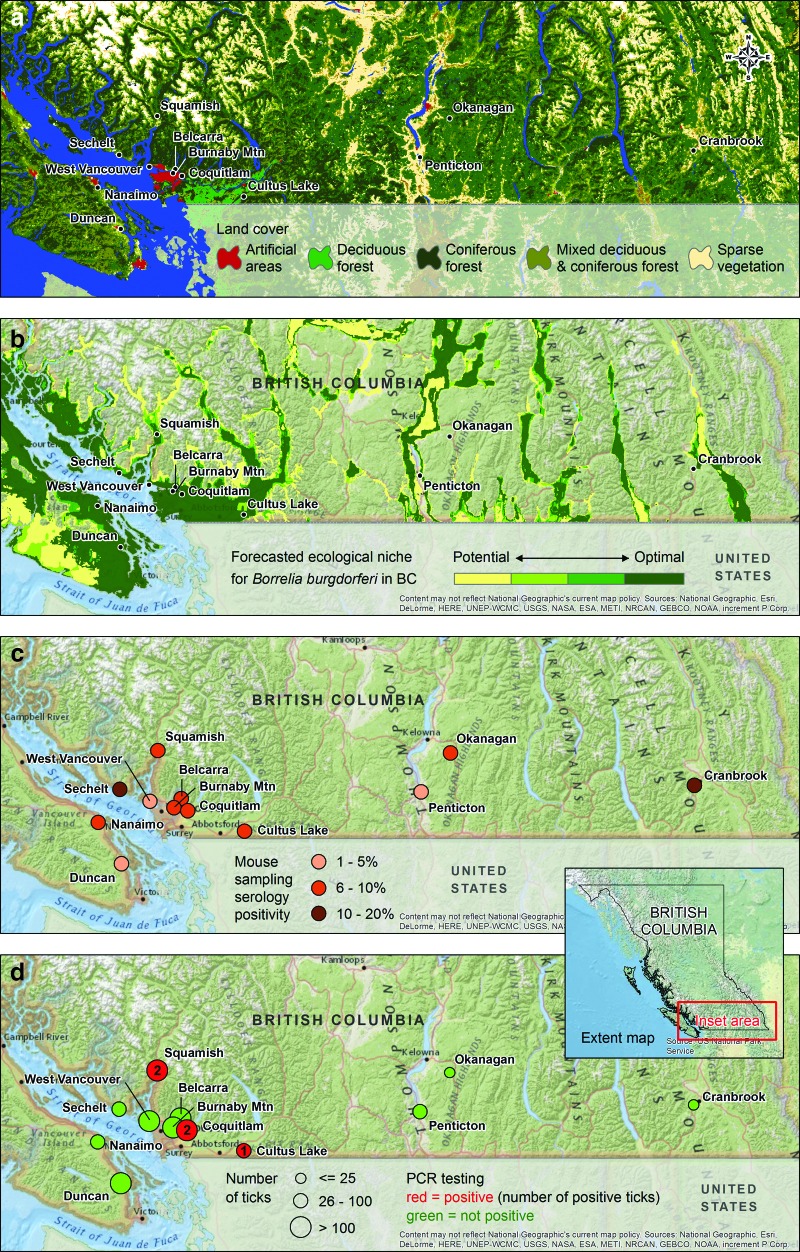
Map of *B. burgdorferi* surveillance in ticks and mice in British Columbia. Land cover type (**a**), ecological niche model for *B. burgdorferi* (**b**), mice (**c**), and ticks (**d**) captured from study locations. The sampling sites of Belcarra, Nanaimo, Squamish, and West Vancouver were dominated with needle leaf coniferous forest (predominantly western hemlock [*Tsuga heterophylla*], Douglas fir [*Pseudotsuga menziesii*], and western red cedar [*Thuja plicata*]); Burnaby Mountain, Cultus Lake, Coquitlam, Duncan, and Sechelt sites were mixed deciduous and coniferous forest (predominantly red alder [*Alnus rubra*], trembling aspen [*Populus tremuloides*], and western hemlock); and Cranbrook, Okanagan, and Penticton sites were sparsely vegetated (low-lying brush or scrub and lodgepole pine [*Pinus contorta*]). Map data sources: BCCDC, European Space Agency (ESA), National Geographic, Esri, DeLorme, HERE, United Nations Environment Programme–World Conservation Monitoring Centre (UNEP-WCMC), US Geological Survey (USGS), National Aeronautics and Space Administration (NASA), Japan Ministry of Economy, Trade and Industry (METI), Natural Resources Canada (NRCan), General Bathymetric Chart of the Oceans (GEBCO), Natinoal Oceanic and Atmospheric Administration (NOAA), and increment P Corp.

Lyme disease is one of the most common vector-borne diseases in North America and its geographic distribution is expanding in eastern Canada (Ogden et al. 2009, Diuk-Wasser et al. 2012). In contrast, Lyme disease prevalence is consistently low in the Pacific Northwest (Henry et al. 2011), possibly due to complex environmental factors, such as unsuitable tick habitat and the predominance of coniferous vegetation (Fig. 1a). We studied the prevalence of *Borrelia burgdorferi* in *Ixodes* spp. ticks (vector) and small rodents (host) in British Columbia for two consecutive years (2013–2014) to determine the risk of human exposure to ticks carrying *B. burgdorferi*.

Samples were collected from 12 sites (Fig. 1c), with the selection of these locations based on a number of factors—previous collection/retrieval of ticks and subsequent isolation of *B. burgdorferi*, predicted ecological niche areas (Fig. 1b) for *B. burgdorferi* (Mak et al. 2010), and proximity to populated areas. Location coordinates were collected using a recreational-grade GPS receiver (eTrex, Garmin, Olathe, KS), and mapped using ArcGIS v10.1 (Esri, Redlands, CA). Past climate trends were examined to identify the optimal timing for tick field surveillance for each location. Appropriate permissions and animal care certificates were obtained from the Ministry of Agriculture, Municipal Park Boards, and the University of British Columbia (UBC) Animal Care committee.

The trapping of rodents and flagging of ticks was conducted between the months of May and August of each year. Small rodents were trapped by deploying 150 Sherman traps (H.B. Sherman Traps, Inc., Tallahassee, FL) (three locations in groups of 50) for two consecutive nights at each site. All captured rodents were euthanized according to UBC animal care–approved protocols. All ticks were removed from the rodents, identified, and subsequently pooled by tick species and life stages to a maximum of five ticks per pool. The surfaces of the mice carcasses were decontaminated with 1:80 Phenokil (Maxim Technologies, Delta, BC), followed by 70% ethanol and rinsing with water. Mouse organs were subsequently harvested using aseptic technique and stored at −80°C until ready for testing. *Ixodes* spp. tick pools and mouse organs (heart and bladder) were tested for the presence of *B. burgdorferi* DNA. Attempts were made to collect ticks by flagging and dragging at each site using white cotton flags (90 cm × 125 cm) by two persons for three consecutive hours, as described previously (Dantas-Torres et al. 2013). Flags were inspected every 5 minutes to ensure no ticks were missed.

DNA from pooled ticks and mouse tissues was extracted according to the QIAGEN Blood and Tissue DNA Extraction Kit protocol (Qiagen, Hilden, Germany). *B. burgdorferi* DNA was detected using real-time PCR and was performed on the Applied Biosystems TaqMan 7500 PCR System (Life Technologies, Burlington, ON). Samples were first subjected to a real-time PCR, targeting the *23S* ribosomal gene for *Borrelia* spp. (including *Borrelia* sensu stricto and sensu lato strains) and subsequently confirmed by a second real-time PCR targeting the *B. burgdorferi* sensu stricto–specific *osp*A gene. Quantitated plasmid controls containing both the *Borrelia* spp. *23S* rRNA and *B. burgdorferi ospA* gene sequences were designed in-house and sent for manufacturing (Integrated DNA Technologies, Mississauga, ON). The cycling conditions for both assays were as follows: Initial denaturation and enzyme activation for 10 min at 95°C, followed by 40 cycles of 95°C for 15 s and 60°C for 1 min (Persing et al. 1990, Postic et al. 1994).

An in-house validated immunofluorescence assay (IFA) was used to determine the presence of *B. burgdorferi* antibodies in mice sera. *B. burgdorferi* B31 strain grown in house in BSK-H media (Sigma Aldrich, Oakville, ON) was diluted to 25 spirochetes per field and fixed to 8-mm-diameter slides. A monoclonal antibody to the *B. burgdorferi* OspA protein (Meridian Life Science, Inc., Memphis, TN) served as a positive control and was used to determine the limit of detection. Goat anti-mouse immunoglobulin G (IgG) antibody conjugated to fluorescein isothiocyanate (FITC; Sigma Aldrich, Oakville, ON) was used to determine the presence of *B. burgdorferi* antibodies in mice sera. Due to the possibility of cross-reactivity of antibodies to other *Borrelia* spp., all equivocal and positive IFA samples were subjected to an in house–validated mice *B. burgdorferi* IgG western blot using the Marblot Strip Test System (Trinity Biotech, Inc., Burlington, ON).

We failed to collect any ticks by dragging/flagging methods, which suggested that either tick density was too low or environmental factors (fauna, temperature, humidity, etc.) were unfavorable for ticks during that time period. Furthermore, our study's focus was to determine the risk of exposure to Lyme disease of the general population; therefore, the chosen study sites were in areas more accessible by the general population rather than in the deep woods where flagging of ticks has been successful previously (unpublished observation). We were not aware of any significant ecological changes at the 12 sample sites since our previous field visit.

Three species of mice and four species of ticks were identified morphologically during this study period. In 2013, 238 small rodents (213 *Peromyscus maniculatus*, 11 *Perognathus parvas*, 13 *Reithrodontomys megalotis*) and one shrew were trapped and a total of 467 ticks (403 *Ixodes pacificus*, 21 *I. angustus*, one *Ixodes* spp., and 42 *Dermacentor andersoni*) were retrieved from those small rodents. Similarly in 2014, 245 small rodents (225 *P. maniculatus*, 19 *P. parvas*) and one shrew were collected and a total of 426 ticks (395 *I. pacificus*, 36 *I. angustus*, two *I. soricis*, one *Ixodes* spp., and 14 *D. andersoni*) were retrieved from the small rodents. Most of the ticks were immature and predominantly larvae (Table 1).

**Table 1.** Detection of *B. burgdorferi* in Ticks Retrieved from Small Rodents in Britisih Colubmia

*Tick Species*	Ixodes Pacificus									Ixodes Angustus									Dermacentor Andersoni									*Grand Total*		
*Tick Stage*	*Adult Female*			*Nymph*			*Larvae*			*Adult Female*			*Nymph*			*Larvae*			*Adult Female*			*Nymph*			*Larvae*			*Each Site*		
*Site Name (Mouse Number)*	*Tick*	*Pool*	B. burgdorferi+	*Tick*	*Pool*	B. burgdorferi+	*Tick*	*Pool*	B. burgdorferi+	*Tick*	*Pool*	B. burgdorferi+	*Tick*	*Pool*	B. burgdorferi+	*Tick*	*Pool*	B. burgdorferi+	*Tick*	*Pool*	B. burgdorferi+	*Tick*	*Pool*	B. burgdorferi+	*Tick*	*Pool*	B. burgdorferi+	*Tick*	*Pool*	B. burgdorferi+
Belcarra (42)	1	1	0	27	13	0	84	30	0	0	0	0	0	0	0	0	0	0	0	0	0	0	0	0	0	0	0	112	44	0
Burnaby Mountain (43)	0	0	0	44	14	0	42	17	0	2	2	0	3	2	0	12	5	0	0	0	0	0	0	0	0	0	0	105	42	0
Coquitlam (46)	1	1	0	18	10	2	89	32	0	0	0	0	0	0	0	0	0	0	0	0	0	2	2	0	0	0	0	110	45	2
Cranbrook (9)	0	0	0	2	2	0	23	5	0	0	0	0	0	0	0	0	0	0	0	0	0	0	0	0	0	0	0	25	7	0
Cultus Lake (40)	0	0	0	10	5	0	22	11	1	0	0	0	0	0	0	0	0	0	0	0	0	0	0	0	0	0	0	32	16	1
Duncan (42)	2	2	0	20	11	0	94	22	0	0	0	0	0	0	0	0	0	0	0	0	0	0	0	0	0	0	0	116	35	0
Nanaimo (49)	3	3	0	18	10	0	27	10	0	0	0	0	2	2	0	1	1	0	0	0	0	0	0	0	0	0	0	52	27	0
Okanagan (28)	0	0	0	0	0	0	0	0	0	0	0	0	0	0	0	0	0	0	2	2	0	11	5	0	11	9	0	24	16	0
Penticton (47)	0	0	0	1	1	0	0	0	0	0	0	0	2	1	0	1	1	0	0	0	0	5	4	0	25	11	0	34	18	0
Sechelt (44)	1	1	0	14	9	0	23	15	0	2	1	0	3	1	0	3	1	0	0	0	0	0	0	0	0	0	0	46	28	0
Squamish (50)	5	4	0	37	12	2	56	20	0	0	0	0	2	2	0	3	1	0	0	0	0	0	0	0	0	0	0	103	39	2
West Vancouver (43)	1	1	0	44	11	0	89	30	0	0	0	0	0	0	0	0	0	0	0	0	0	0	0	0	0	0	0	134	42	0
Grand Total (483)	14	13	0	235	98	4	549	192	1	4	3	0	12	8	0	20	9	0	2	2	0	18	11	0	36	20	0	893	359	5

^a^ One shrew from Cranbrook and one shrew from Cultus Lake were trapped and tested negative for *B. burgdorferi*.

No *B. burgdorferi* DNA was detected in mice tissues (*n* = 483 × 2); however, three out of 186 tick pools in 2013 and two out of 173 tick pools in 2014 were positive for *B. burgdorferi* (Fig. 1d). Of the positive tick pools, two out of five corresponding mice were also serologically positive (Fig. 1c). A subset of blinded tick pool DNA samples (2013, *n* = 48; 2014, *n* = 44), which included the five positive tick pools, was sent to the Centers for Disease Control and Prevention, in Fort Collins, CO, and identical results were produced using two different multiplex real-time PCR assays as previously described (Hojgaard et al. 2014). The two TaqMan PCR multiplex assays have *B. burgdorferi* primers and probes for flagellar filament cap (*fliD)* and for genomic DNA (*gB31)*. This assay also detected *Anaplasma phagocytophilum* and *Babesia microti*, but none of these pathogens were found in the samples analyzed with this assay (Table 1). Locations with positive findings were Coquitlam, Squamish, and Cultus Lake (Fig. 1c); all sites had *B. burgdorferi*–positive ticks in the past.

Nineteen mice were serologically confirmed to have antibodies against *B. burgdorferi* in 2013 and 22 mice in 2014 (Table 2). The prevalence of *B. burgdorferi* in the British Colubmia *Ixodes* tick population and 95% confidence interval (CI) were estimated to be 0.56% (0.01%, 1.18%), which is very low. The exposure rate in the mice population to *B. burgdorferi* and 95% CI were estimated to be 8.49% (5.47%, 12.60%), which suggested that the infestation rate is also low in the host population (Fig. 1c). In a previous study, we tested 3500 deer mice by culture and found 30 (0.83%) positive (98). At the same time, we also tested 164 deer mice for antibodies to *B. burgdorferi* and found 6 (3.66%) positive, which demonstrated a low prevalence in this reservoir.

**Table 2.** Prevalence of *B. burgdorferi* Antibodies in Small Rodent's Sera Captured from Different Locations in British Columbia

*Mouse Species*	Peromyscus Maniculatus		Perognathus Parvus		Reithrodontomys Megalotis		*Grand Total*	
*Site (Mouse Number)*	*Total Number*	*Seropositive number*	*Total Number*	*Seropositive number*	*Total Number*	*Seropositive number*	*Total Number*	*Seropositive number*
Belcarra (42)	42	4	0	0	0	0	42	4
Burnaby Mountain (43)	43	4	0	0	0	0	43	4
Coquitlam (46)	46	4	0	0	0	0	46	4
Cranbrook (9)	8	1	0	0	0	0	9	1
Cultus Lake (40)	39	4	0	0	0	0	40	4
Duncan (42)	42	2	0	0	0	0	42	2
Nanaimo (49)	49	3	0	0	0	0	49	3
Okanagan (28)	10	0	18	2	0	0	28	2
Penticton (47)	35	2	12	0	0	0	47	2
Sechelt (44)	37	6	0	0	7	3	44	9
Squamish (50)	44	3	0	0	6	2	50	5
West Vancouver (43)	43	1	0	0	0	0	43	1
Grand Total (483)	438	34	30	2	13	5	483	41

^a^ Two *I. soricis* (one adult female and one nymph) found on Burnaby Mountain and one adult female *Ixodes* spp. from Nanaimo were tested PCR negative.

The British Colubmia Public Health Microbiology and Reference Laboratory (BCPHMRL) has been actively testing ticks for *B. burgdorferi* in over 125 areas of British Columbia since 1993. From 1993 to 1996, 10,056 ticks were tested with a prevalence of 0.40%; 8602 ticks were tested in 1997 to 2007, with a prevalence of 0.35% demonstrating a stable, low prevalence of infection (Henry and Morshed 2011). Eleven of the 12 current sites were visited in 2004, where a total of 218 *P. maniculatus* mice were trapped and 722 immature *I. pacificus* were retrieved from these mice. No rodent tissues or retrieved ticks were positive by culture or PCR, which suggested that rodents were not infected or infective at the time of capture even though seropositive mice were found. BCPHMRL provides serological testing of Lyme disease as part of its clinical diagnostic services. Data from 1997 to 2008 indicated three to 15 cases reported annually, in which 33% of the cases acquired the infection out of province. Moreover, the annualized case rate of Lyme disease in the Pacific Northwest, specifically in British Columbia, Washington State (both 0.19/100,000), and California (0.26/100,000), were dramatically lower than that of northeastern North America, namely, Connecticut (84.43/100,000), during the same time period (Henry et al. 2011).

Our current observation of a continual, low prevalence of *B. burgdorferi* is consistent with historical data from active surveillance of tick populations in British Colubmia (Henry and Morshed 2011), and from Lyme disease case data from neighboring states Washington, Idaho, and Oregon (Centers for Disease Control and Prevention 2015). The differences in vector and host species between eastern and western Canada, complex environmental factors (flora and fauna), and uncertainty regarding the seasonal timing of tick life cycles may contribute to this lowered prevalence of *B. burgdorferi* in British Columbia and the Pacific Northwest.
